# Ambulatory Thoracoscopic Pleurodesis Combined With Indwelling Pleural Catheter in Malignant Pleural Effusion

**DOI:** 10.3389/fsurg.2021.738719

**Published:** 2021-10-25

**Authors:** Chuan T. Foo, Thomas Pulimood, Martin Knolle, Stefan J. Marciniak, Jurgen Herre

**Affiliations:** ^1^Cambridge University Hospitals National Health Service Foundation Trust, Cambridge, United Kingdom; ^2^Cambridge Institute for Medical Research, University of Cambridge, Cambridge, United Kingdom

**Keywords:** neoplasia, outpatient, pleurodesis, pleural effusion, malignant, thoracoscope

## Abstract

**Background and Objective:** Malignant pleural effusion (MPE) often results in debilitating symptoms. Relief of dyspnoea and improvement in quality of life can be achieved with either talc pleurodesis or insertion of an indwelling tunneled pleural catheter (IPC). The former requires a lengthy hospital stay and the latter is associated with lower pleurodesis rates. In response to limited hospital bed capacity, we developed a pragmatic approach in managing MPE by combining thoracoscopic talc poudrage and insertion of IPC into a single day case procedure. We present data on the safety and efficacy of this approach.

**Methods:** Patients who had undergone the abovementioned procedure between 2017 and 2020 were analyzed. Demographic data, hospital length of stay (LOS), histological diagnosis, rates of pleurodesis success and procedural related complications were collated. Patients were followed-up for 6 months.

**Results:** Forty-five patients underwent the procedure. Mean age was 68.5 ± 10.4 years and 56% were male. Histological diagnosis was achieved in all cases. 86.7% of patients were discharged on the day of the procedure. Median LOS was 0 (IQR 0–0) days. Successful pleurodesis was attained in 77.8% at 6-month follow-up. No procedure related deaths or IPC related infections were recorded.

**Conclusion:** Ambulatory thoracoscopic poudrage and IPC insertion is a safe and effective option in the management of MPE. All patients received a definitive pleural intervention with 77.8% pleurodesis success at 6-months and majority of them discharged on the same day. Future randomized trials are required to confirm these findings.

## Introduction

Malignant pleural effusion (MPE) affects up to 15% of patients with cancer (1). MPE is usually the result of malignant infiltration of the pleural and commonly causes debilitating symptoms such as dyspnoea, cough, and chest pain. The goal in managing MPE is to relieve dyspnoea and improve quality of life (2). This is often achieved with chemical pleurodesis using medical graded talc, or an indwelling tunneled pleural catheter (IPC). Talc pleurodesis is highly effective but requires 4–6 days hospitalization (3, 4). In contrast, IPCs are routinely inserted as day-case procedures however confer a much lower pleurodesis rate even when used in combination with talc (5, 6). Furthermore, there is an inherent risk of infection with IPCs (7), as well as the cost and inconvenience associated with community drainages (8).

In response to the increasing number of patients with MPE seen at our institution and the challenges with hospital bed capacity, we developed a pragmatic approach in the diagnosis and management of MPE by combining medical thoracoscopy with talc poudrage and insertion of IPC into a single day case procedure. In doing so, we take advantage of both management strategies while minimizing their disadvantages.

In this paper, we retrospectively describe our experience and outcomes of this approach.

## Methods

### Patients

In this retrospective chart-based study, patients who had undergone the above-mentioned procedure between March 2017 and March 2020 for diagnosis or management of a suspected or proven MPE were identified. Patients were ≥18 years old, had an Eastern Cooperative Oncology Group performance status of ≤2, an expected survival of >2 months, and judged to be able to tolerate the procedure under conscious sedation. Lung re-expansion was confirmed on chest x-ray (CXR) post-therapeutic aspiration in 93% (42/45) of patients. The remaining 7% (3 patients) underwent the procedure upfront. Clinical data analysis was approved by Addenbrooke's Hospital Institutional Review Board (ID3673).

### Procedure

Patients underwent ultrasound guided rigid thoracoscopy with a single 7 mm port in a standard bronchoscopy suite by a pulmonologist. All patients received midazolam for sedation, fentanyl for analgesia, and local anesthesia. Pleural fluid was evacuated before biopsies were performed followed by placement of a Rocket® IPC via a new incision. Steritalc® (Novatech SA, France) 3 g was insufflated into the pleural cavity. A 20 Fr intercostal drain was placed through the port site and connected to 4 kPa suction. The IPC was not used in the immediate post-procedure period. Patients were transferred to the recovery area for monitoring and a CXR was performed 1–2 h after the procedure. Intercostal drain was removed if CXR showed resolution of procedure-induced pneumothorax and no clinical evidence of air leak. Patients were discharged if clinically stable with follow-up at their local center. IPC was drained daily for the first 2 days and thrice a week subsequently or as determined by the treating clinician. All patients also had a routine clinical review 2–3 days after their procedure, with any further appointments at the discretion of the local team or at the patient's request, usually only if there are concerns with the IPC e.g., low or no output, pain, suspected blockage or infection. Patients were followed for 6 months post-procedure.

### Data Collected

Data collected included demographics, hospital length of stay (LOS), histological diagnosis, amount of procedural sedation, and rates of successful pleurodesis at 3- and 6-months. Patients' survival status were determined from medical records or telephone call 6 months post-procedure. Successful pleurodesis was defined as the removal of the IPC which occurred if <50 ml of fluid was drained on three consecutive occasions and CXR showed <25% opacification of the appropriate hemithorax due to suspected fluid. Thoracic ultrasonography was used to assess the absence of lung sliding and confirm CXR findings. Patients requiring aspiration of >100 ml of fluid; chest drain or IPC insertion for fluid management; and thoracoscopy of any kind on the same side as the IPC after its removal, or who declined or died before these procedures could be performed were classified as failed pleurodesis. Procedural complications and IPC related infections were noted. Data were summarized using mean, median, standard deviation and interquartile ranges, or number (%). Six-month survival was calculated using the Kaplan-Meier method. Statistical analyses were performed using IBM® SPSS® Statistics 26.

## Results

Forty-five patients were included in the study. Mean age was 68.5 ± 10.4 years and 25 (56%) were male. An average of 3.7 ± 1.3 mg of midazolam and 101.7 ± 26.9 mcg of fentanyl were required for procedural sedation and analgesia. Pleural malignancy was histologically confirmed in all but one patient who had fibrinous pleuritis. The most common malignancy was mesothelioma (*n* = 10), followed by lung (*n* = 9) and breast cancer (*n* = 9). Thirty-nine (86.7%) patients spent a mean of 298.9 ± 96.7 min in recovery prior to discharge. Six (13.3%) required admission for the following reasons: 3 (50%) persistent air leak; 2 (33.3%) hypotension; 1 (16.7%) pneumonia. Median LOS was 0 (IQR 0–0) days, and ranged from 1 to 9 days with a median of 2.5 (IQR 1–5.5) days in those admitted (Table 1).

**Table 1:** Patient characteristics and summary of results.

**Characteristics**	**Value**
**Demographics**	
Age (years)	68.5 ± 10.4
Male	25 (55.6)
Female	20 (44.4)
**Sedation/Analgesia**	
Midazolam (mg)	3.7 ± 1.3
Fentanyl (mcg)	101.7 ± 26.9
**Histological diagnosis**	45
Mesothelioma	15 (33.3)
Lung	9 (20)
Breast	9 (20)
Ovarian	5 (11)
Genitourinary	4 (8.9)
Gastrointestinal	1 (2.2)
Melanoma	1 (2.2)
Fibrinous pleuritis	1 (2.2)
**Total cases**	45
Day case	39 (86.7)
Required admission	6 (13.3)
Air leak	3 (50)
Hypotension	2 (33.3)
Pneumonia	1 (16.7)
**Length of stay (days)**	
Overall	0 (IQR 0–0)
Admitted cases	2.5 (IQR 1–5.5)
**Pleurodesis success**	
3 months	32 (71.1)
6 months	35 (77.8)

*Values are presented as mean ± standard deviation, median (IQR), or number (%)*.

**Figure 1:**
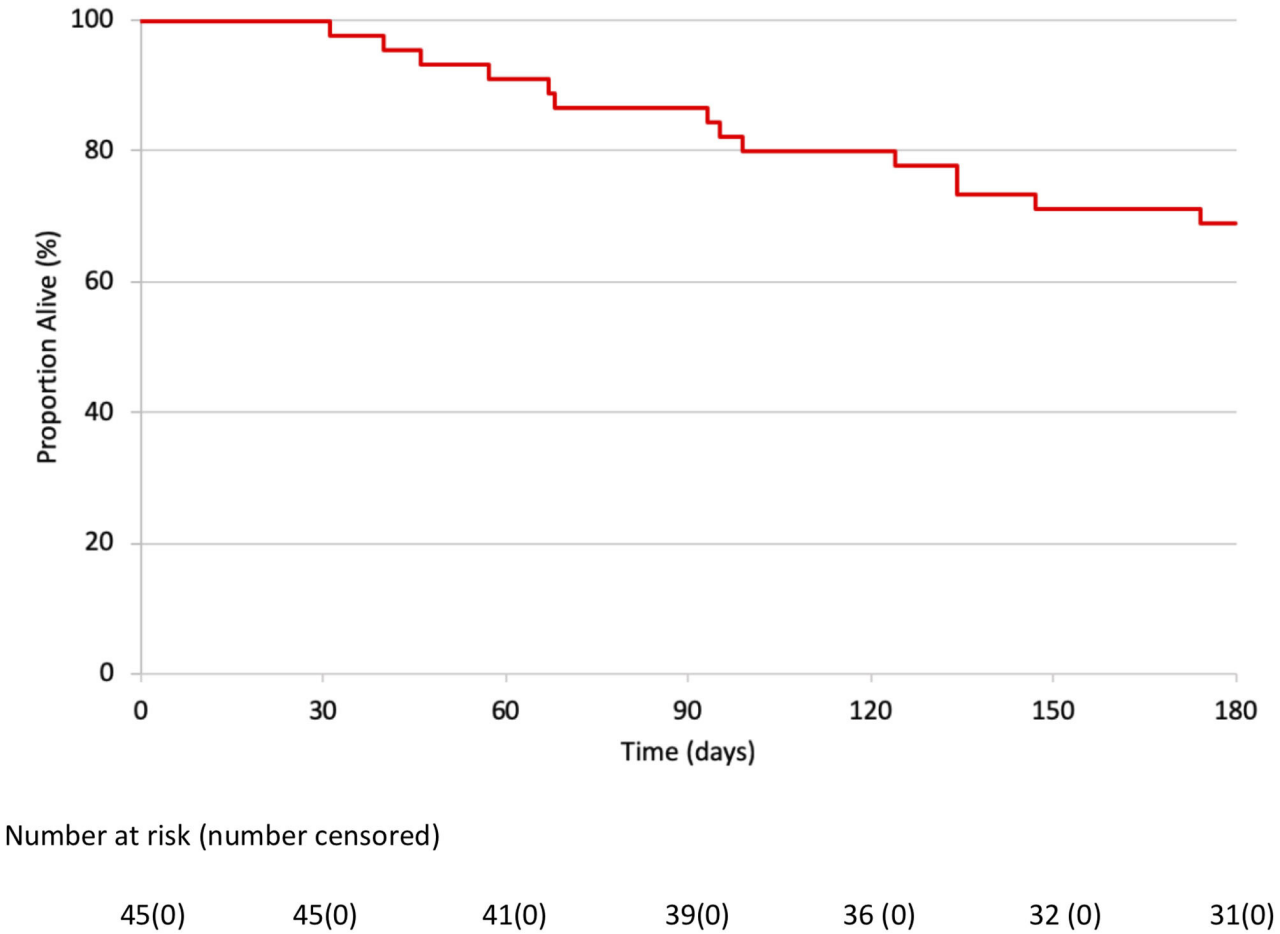
Kaplan Meier survival curve of 45 patients after combined thoracoscopic procedure.

IPC was removed at a median of 20 (IQR 13–48) days. Successful pleurodesis was achieved in 32 (71.1%) patients at 3-month and 35 (77.8%) patients at 6-month follow-up. Between 3- and 6-months, one patient who had previously achieved pleurodesis had a recurrence of the pleural effusion and was re-classified as pleurodesis failure. In the same time period, a further 4 patients had achieved successful pleurodesis (Table 1). Fourteen (31.1%) patients died from progression of malignancy within 6 months post-procedure (Figure 1). There were no procedure related deaths or IPC related complications (infections, blockages, dislodgements, or catheter fractures on removal).

## Discussion

Current guidelines recommend talc pleurodesis or IPC in the management of MPE (1, 2). While the recent TAPPS trial showed talc poudrage to be similar in efficacy to talc slurry in achieving pleurodesis (78 vs. 76% at 90 days, 71 vs. 72% at 180 days), mean LOS was 5–6 days (3). Conversely, IPCs are routinely inserted in an outpatient setting and while effective at relieving dyspnoea, achieve much lower auto-pleurodesis rates despite addition of talc (43 vs. 23% at 35 days), or aggressive drainage (47 vs. 24% at 12 weeks) (5, 9). Additional disadvantages of an IPC-centric strategy include the inconvenience of community drainages, risk of IPC related infections, and cost of drainage bottles (10).

In this study, we demonstrated a safe and effective approach in the ambulatory management of MPE by combining thoracoscopic poudrage and IPC insertion into a single day-case procedure. Our pleurodesis success rates of 71.1 and 78.8% at 3 and 6-months compare well with TAPPS, and are higher than in trials utilizing IPCs. Furthermore, 86.7% of patients were safely discharged on the same day, reducing reliance on hospital bed capacity which may be severely limited in situations such as the coronavirus pandemic. The short LOS is also likely to be important to patients given the limited median survival for individuals with MPE (1). Importantly, all patients also received a definitive pleural intervention.

To our knowledge, only three other studies have examined combination strategies similar to ours. In a pilot study of 30 patients, Reddy et al. described a rapid pleurodesis protocol and demonstrated a 3.19-day mean hospital length of stay with a 92% pleurodesis success rate at 6 months and universal improvement in dyspnoea and quality of life scores (11). A subsequent retrospective review of an additional 29 patients who underwent the same rapid pleurodesis protocol after the initial trial ended recorded a median LOS of 2 days and a successful pleurodesis rate of 79% although the duration of follow-up was unclear (12). A separate small study by Boujaoude et al. involving 29 patients reported a 92% pleurodesis success rate at 1 month and a median duration of hospitalization of 3 days as well as improvement in dyspnoea scores (13). Compared to our approach, patients in these three studies were routinely admitted post-procedure and underwent aggressive drainage of their IPC (7 drainages in the first 3 days followed by daily drainage in Reddy et al., and daily drainages in Krochmal et al. and Boujaoude et al.), a resource intensive and burdensome process. Crucially, patients who died were excluded from the final analysis. Nevertheless, these findings support the safety and efficacy of combination approaches.

Our study was limited by its retrospective design and small sample size. Moreover, despite using ultrasound to assess for evidence of pleurodesis, a validated protocol such as that published by Chaddha et al. was not utilized (14). Consequently, the possibility that the resolution of effusion was due to disease control could not be excluded and future trials should be designed to address these factors. Thirdly, although all patients reported subjective improvement in dyspnoea and quality of life at follow-up, these were not objectively measured.

## Conclusion

This study highlights the safety, feasibility, and efficacy of a pragmatic one-stop approach in the diagnosis and management of MPE. Future randomized trials comparing this approach with standard talc pleurodesis are required to confirm these findings. Health economic data will also be of interest.

## Data Availability Statement

The raw data supporting the conclusions of this article will be made available by the authors, without undue reservation.

## Ethics Statement

The studies involving human participants were reviewed and approved by Addenbrooke's Hospital Institutional Review Board (ID3673). The patients/participants provided their written informed consent to participate in this study.

## Author Contributions

TP, MK, SM, and JH: conceptualization. CF, TP, MK, and JH: data curation. CF, TP, and JH: formal analysis. CF: writing of original draft. CF, TP, MK, SM, and JH: review and approval of final version. All authors contributed to the article and approved the submitted version.

## Conflict of Interest

The authors declare that the research was conducted in the absence of any commercial or financial relationships that could be construed as a potential conflict of interest.

## Publisher's Note

All claims expressed in this article are solely those of the authors and do not necessarily represent those of their affiliated organizations, or those of the publisher, the editors and the reviewers. Any product that may be evaluated in this article, or claim that may be made by its manufacturer, is not guaranteed or endorsed by the publisher.

## References

[1] Bibby AC Dorn P Psallidas I Porcel JM Janssen J Froudarakis M. ERS/EACTS statement on the management of malignant pleural effusions. Eur J Cardiothorac Surg. (2019) 55:116–32. 10.1093/ejcts/ezy258 30060030

[2] Feller-Kopman DJ Reddy CB DeCamp MM Diekemper RL Gould MK Henry T. Management of malignant pleural effusions. An official ATS/STS/STR clinical practice guideline. Am J Respir Crit Care Med. (2018) 198:839–49. 10.1164/rccm.201807-1415ST 30272503

[3] Bhatnagar R Piotrowska HEG Laskawiec-Szkonter M Kahan BC Luengo-Fernandez R Pepperell JCT. Effect of thoracoscopic talc poudrage vs. talc slurry via chest tube on pleurodesis failure rate among patients with malignant pleural effusions: a randomized clinical trial. JAMA. (2020) 323:60–9. 10.1001/jama.2019.19997 31804680 PMC6990658

[4] Davies HE Mishra EK Kahan BC Wrightson JM Stanton AE Guhan A. Effect of an indwelling pleural catheter vs chest tube and talc pleurodesis for relieving dyspnea in patients with malignant pleural effusion: the TIME2 randomized controlled trial. JAMA. (2012) 307:2383–9. 10.1001/jama.2012.5535 22610520

[5] Bhatnagar R Keenan EK Morley AJ Kahan BC Stanton AE Haris M. Outpatient talc administration by indwelling pleural catheter for malignant effusion. N Engl J Med. (2018) 378:1313–22. 10.1056/NEJMoa1716883 29617585

[6] Van Meter ME McKee KY Kohlwes RJ. Efficacy and safety of tunneled pleural catheters in adults with malignant pleural effusions: a systematic review. J Gen Intern Med. (2011) 26:70–6. 10.1007/s11606-010-1472-0 20697963 PMC3024099

[7] Fysh ETH Tremblay A Feller-Kopman D Mishra EK Slade M Garske L. Clinical outcomes of indwelling pleural catheter-related pleural infections: an international multicenter study. Chest. (2013) 144:1597–602. 10.1378/chest.12-3103 23828305

[8] Olfert JA Penz ED Manns BJ Mishra EK Davies HE Miller RF. Cost-effectiveness of indwelling pleural catheter compared with talc in malignant pleural effusion. Respirology. (2017) 22:764–70. 10.1111/resp.12962 27983774

[9] Wahidi MM Reddy C Yarmus L Feller-Kopman D Musani A Shepherd RW. Randomized trial of pleural fluid drainage frequency in patients with malignant pleural effusions. The ASAP trial. Am J Respir Crit Care Med. (2017) 195:1050–7. 10.1164/rccm.201607-1404OC 27898215

[10] Fortin M Tremblay A. Pleural controversies: indwelling pleural catheter vs. pleurodesis for malignant pleural effusions. J Thorac Dis. (2015) 7:1052–7. 10.3978/j.issn.2072-1439.2015.01.51 26150918 PMC4466431

[11] Reddy C Ernst A Lamb C Feller-Kopman D. Rapid pleurodesis for malignant pleural effusions: a pilot study. Chest. (2011) 139:1419–23. 10.1378/chest.10-1868 20930006

[12] Krochmal R Reddy C Yarmus L Desai NR Feller-Kopman D Lee HJ. Patient evaluation for rapid pleurodesis of malignant pleural effusions. J Thorac Dis. (2016) 8:2538–43. 10.21037/jtd.2016.08.55 27747006 PMC5059296

[13] Boujaoude Z Bartter T Abboud M Pratter M Abouzgheib W. Pleuroscopic pleurodesis combined with tunneled pleural catheter for management of malignant pleural effusion: a prospective observational study. J Bronchol Interv Pulmonol. (2015) 22:237–43. 10.1097/LBR.0000000000000186 26165894

[14] Chaddha U Agrawal A Bhavani SV Sivertsen K Donington DJ Ferguson MK. Thoracic ultrasound as a predictor of pleurodesis success at the time of indwelling pleural catheter removal. Respirology. (2021) 26:249–54. 10.1111/resp.13937 32929838

